# The urban physical environment and leisure-time physical activity in early midlife: a FinnTwin12 study

**DOI:** 10.1016/j.healthplace.2025.103495

**Published:** 2025-06-04

**Authors:** Zhiyang Wang, Sari Aaltonen, Roos Teeuwen, Vasileios Milias, Carmen Peuters, Bruno Raimbault, Teemu Palviainen, Erin Lumpe, Danielle Dick, Jessica E. Salvatore, Maria Foraster, Payam Dadvand, Jordi Júlvez, Achilleas Psyllidis, Irene van Kamp, Jaakko Kaprio

**Affiliations:** a Institute for Molecular Medicine Finland, Helsinki Institute of Life Science, University of Helsinki, Helsinki, Finland; b Department of Sustainable Design Engineering, Delft University of Technology, Delft, the Netherlands; c ISGlobal, Barcelona Biomedical Research Park (PRBB), Barcelona, Spain; d Universitat Pompeu Fabra (UPF), Barcelona, Spain; e Spanish Consortium for Research on Epidemiology and Public Health (CIBERESP), Instituto de Salud Carlos III, Madrid, Spain; f Department of Psychology, Rutgers University, New Brunswick, NJ, USA; g Department of Psychiatry, Rutgers University, Piscataway, NJ, USA; h PHAGEX Research Group, Blanquerna School of Health Science, Universitat Ramon Llull (URL), Barcelona, Spain; i Clinical and Epidemiological Neuroscience (NeuroÈpia), Institut d’Investigació Sanitària Pere Virgili (IISPV), Reus, Spain; j National Institute for Public Health and the Environment, the Netherlands

**Keywords:** Exercise, Machine learning, Urbanization, Behavior

## Abstract

Under the exposome framework, this study examined the relationship between the urban physical environment and leisure-time physical activity during early midlife based on 394 participants (mean age: 37, range 34–40) from the FinnTwin12 cohort, residing in five major Finnish cities in 2020. We curated 145 urban physical exposures based on residential addresses and measured three outcomes: total leisure-time physical activity (total LTPA) and two sub-domains: leisure-time physical activity without commuting activity (LTPA) and commuting activity. K-prototypes clustering identified three urban clusters: “original city center,” “new city center,” and “suburban,” each with distinct environmental patterns. Regression models showed that participants in the “suburban” cluster had lower levels of total LTPA and LTPA compared to those in the “original city center” cluster, while we found null findings for commuting activity. Then, repeated regression models with a p-value threshold of 0.01 were used to initially select candidates. eXtreme Gradient Boosting models identified greenspaces and road characteristics as the top important factors influencing total LTPA, while pocket park and greenness were ranked as the top important factors influencing LTPA. The relationships were non-linear. There were thresholds for the count and size of pocket parks within 800 m walking distance and the modified soil adjusted vegetation index, determining whether they positively or negatively predict LTPA. Our findings suggested that the urban environment in Finnish cities was associated with leisure-time physical activity, which revealed new residential pattern and identified key exposures of road, pocket park, and greenness with non-linear effect, that can guide future policies.

## 1. Introduction

Regular physical activity has been widely demonstrated to prevent multiple non-communicable diseases and reduce the risk of premature death (Booth et al., 2012). The economic and health burden arising from physical inactivity is substantial and continually rising, costing public health care systems an estimated USD 47.6 billion globally every year (Santos et al., 2023). Since previous studies show a strong contribution of environmental factors to physical activity (Boomsma et al., 2011; Carlin et al., 2017; Duncan et al., 2008), interventions targeting the environment may be a good entry point to promote physical activity. Early midlife is a crucial but relatively overlooked stage for investigating physical activity, as it reflects established long-term health behavior patterns and marks the period between key transitions in young adulthood and later life. Unhealthy behaviors during this stage can increase the risk of non-communicable diseases (Dagenais et al., 2021), and a recent study showed that early midlife physical activity strongly predicts future behavior (Berntzen et al., 2024).

Urbanization stands as a transformative trend, with more than half the world’s population currently residing in urban areas (United Nations Human Settlements Programme, 2022). Many reviews have summarized the salient link between the urban environment and physical activity (Ding et al., 2011; Durand et al., 2011; Kärmeniemi et al., 2018). The exposome offers a theoretical framework with an umbrella perspective to depict the totality of the environment that people experience (Wild, 2012) and examine health effects from the real-world urban environment, of which the urban physical component plays an important role. The exposome studies have the potential to unveil more comprehensive non-genetic predictors through large-scale characterization of the environment. Gorman et al. (2021) have outlined the bidirectional effect between the exposome and physical activity but pointed out the uncertainty in mechanisms and interactions. The urban physical exposome is ubiquitous and multifaceted, which makes it a complex entity to study.

Every environmental factor contributes to this complex totality of exposures, and no factor is isolated. Urban regeneration projects are a good example, usually designed to improve public health by implementing structural and risk-minimizing solutions. They often yield collateral effects on other aspects, such as bringing economic, social, and cultural benefits, within the city’s complex system (Chen et al., 2023; Sonnenschein et al., 2022; Wang et al., 2021). For example, an urban riverside park regeneration project in Barcelona, Spain was estimated to attract over five thousand adult users daily to perform different types of physical activity (Vert et al., 2019). Beyond the project’s basic objectives, an open-air museum will be built there, transforming social and built environments. Nowadays, regeneration projects around the world are often multi-component and intersectoral. In another Barcelona regeneration program, aiming to improve living conditions in the most disadvantaged neighborhoods (involving, for example, social services, greenspaces, and household support), researchers found that the neighborhood with a bigger project budget was associated with a higher frequency of physical activity among residents (Bartoll-Roca et al., 2024). Previous studies relying on single exposures or limited sets were relatively inadequate to depict the broader urban environment and its health effects.

Several approaches have been developed to address the challenges of high-dimensional exposome data. Feature extraction involves transforming numerous exposures into informative and compact indices. One such method, clustering analysis, provides insights into real-world scenarios and offers high scalability to uncover hidden patterns instead pre-specified classification. Another approach involves machine learning models, widely used in exposome research, which surpass conventional analyses by capturing non-linear relationships, disentangling interactions, and providing robust computation for multi-inference (Ohanyan et al., 2022). This approach may deepen the understanding of the distinct and complex characteristics of the urban physical exposome. Related to the urban physical exposome and physical activity, previous studies have applied such an approach, however, the number of exposures included in those studies (Lee et al., 2023; Ping et al., 2023; Zang et al., 2022) were small (6–28), restricting their ability to capture the complexity of the urban exposome. In addition, research linking detailed exposure profiling and leisure-time physical is also limited in Finland and Nordic countries.

In this study, we adopted a holistic perspective to examine the association between the urban physical component of the exposome and leisure-time physical activity during early midlife, moving beyond the conventional “one exposure” epidemiological model. There were two key objectives. The first was to detect people with heterogeneous urban living environments using cluster analysis and to compare leisure-time physical activity levels between clusters. The second objective was to rank urban physical exposures by importance with respect to leisure-time physical activity, to examine non-linear relationships, and to detect pairwise interactions between exposures by the gradient boosting method. The aim was to provide actionable evidence for local policymakers and other stakeholders about the complex effect of the urban environment on leisure-time physical activity. This would facilitate the development of precise and cost-effective interventions to address the physical inactivity challenge.

## 2. Material and methods

**Fig. 1.**
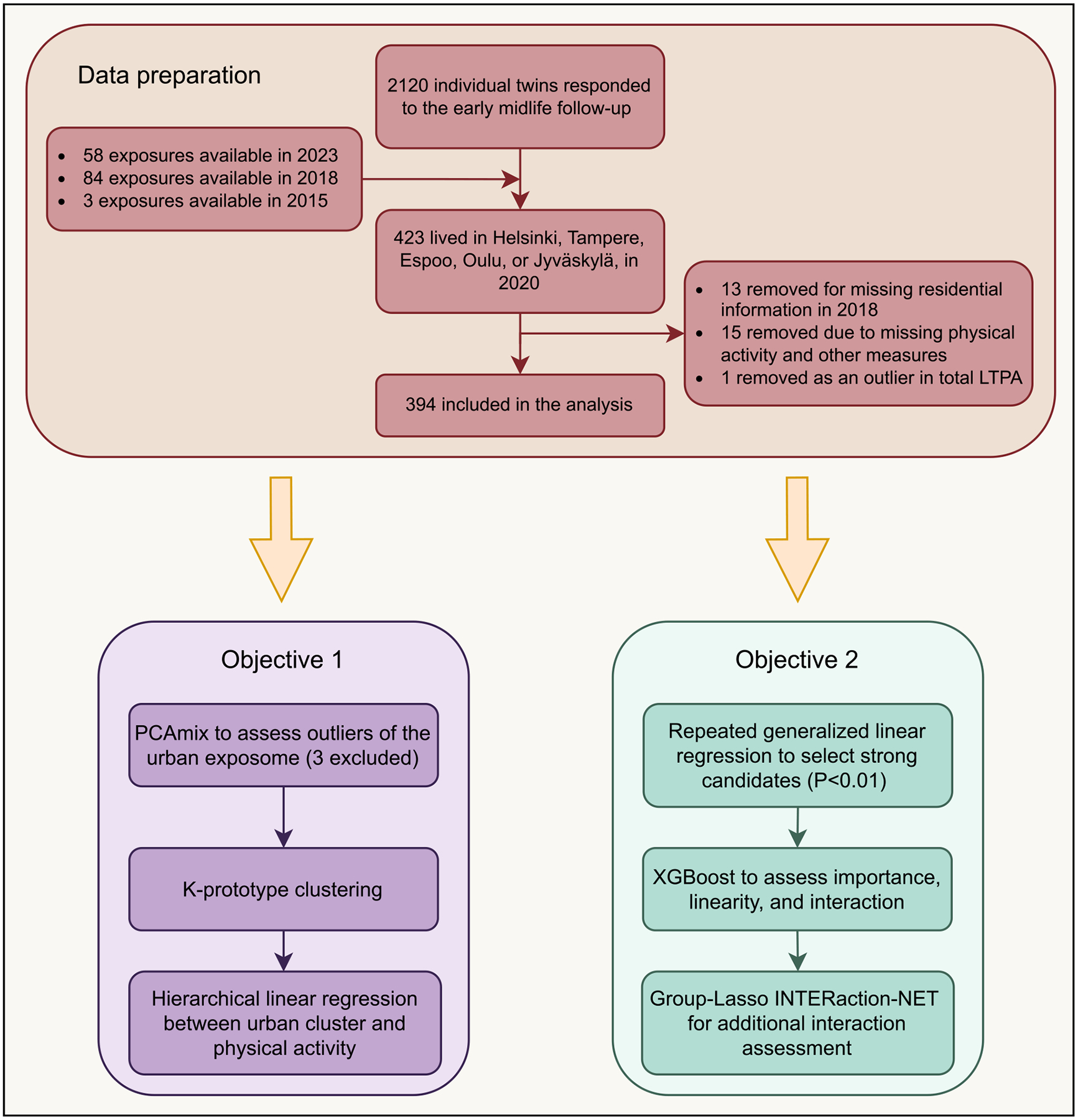
Study flow.

The flow chart of this study is presented in Fig. 1.

### 2.1. Participants

Participants were from the FinnTwin12 cohort, a nationwide prospective cohort of all Finnish twins born between 1983 and 1987, identified and collected from the nation’s Central Population Registry. Briefly, at baseline (1994–1999), 5522 12-year-old twins were invited to participate and 87 % of them agreed to take part. There were four follow-ups: age 14, age 17, young adulthood (mean age 24), and early midlife (mean age 37), with retention rates of 92 %, 75 %, 66 %, and 41 %, respectively. A recent study has detailed the latest follow-up of the cohort (Cooke et al., 2025). In this study, we included individual twins who lived in five large cities of Finland, namely Helsinki, Tampere, Espoo, Oulu, or Jyväskylä, in 2020, resulting into a smaller sample in the present analysis. According to the Statistics Finland, 11.9 %, 4.4 %, 5.3 %, 3.7 %, and 2.6 % of the total population resided in Helsinki, Tampere, Espoo, Oulu, and Jyväskylä in 2020, respectively.

### 2.2. Measures

#### 2.2.1. Leisure-time physical activity

Our study focuses on early midlife leisure-time physical activity, which is performed at the person’s discretion, along with essential daily living activities or work-related tasks (Caspersen et al., 1985). This type of physical activity is considered one of the most effective ways to increase overall physical activity levels (Borodulin et al., 2008). It was measured through structured and validated questions on the frequency, mean duration, and mean intensity of participants’ leisure-time physical activity sessions, as well as a question on their commuting activity (Leskinen et al., 2009; Waller et al., 2008). Based on these structured questions, we quantified mean metabolic equivalent of task (MET) hours per day, which expressed the energy cost of physical activities in the form of the resting metabolic rate (Jetté et al., 1990), as the unit of physical activity level. The calculation formula for the mean MET hours per day was the following: physical activity frequency (average per day) × physical activity duration (average hours) × physical activity intensity (physical activity MET value) (Kujala et al., 1998). The MET values for activity intensity were: 4 for intensity corresponding to walking, 6 for vigorous walking to jogging, 10 for jogging, and 13 for running. All types of leisure-time physical activities were considered when MET hours per day were calculated. We assumed that commuting activity was done on 5 days per week and on the intensity of walking. The questions are listed in Supplemental Note 1 and the comparison of MET specification between the Compendium of Physical Activity and this study is presented in Supplementary Table 1.

The primary measure, *total leisure-time physical activity (total LTPA)*, was the sum of two sub-domains (secondary measures): 1) *leisure-time physical activity without commuting activity (LTPA)* and 2) *commuting activity*. All these measures were expressed as the mean MET hours per day. The higher level of MET hours per day means the higher the level of activity. Participants with over mean 45 MET hours/day of total LTPA were identified as outliers and removed. This threshold corresponds to, for example, approximately 3.5 h of fast running daily, which is likely unrealistic (Ainsworth et al., 2011; Herrmann et al., 2024). The distributions of all three measures are shown in Supplemental Fig. 1, and due to the skewness, we log-transformed them.

#### 2.2.2. Urban physical exposome

We assigned 145 indicators of urban physical exposures to the residential address of each study participant. Detailed description and summary statistics of these indicators are presented in Supplemental Table 2. The urban physical exposome set comprehensively depicted the urban environment including aspects such as traffic, streets, land use, green (i.e. parks, forests, and fields) and blue (i.e. lakes and seas) spaces, and so on. The computing and enriching process was on the geocode level and derived from multiple open sources, described in Supplemental Note 2 and elsewhere (Milias and Psyllidis, 2021; Teeuwen et al., 2023; van Kamp et al., 2022; Wang et al., 2023). Most urban physical exposures were measured or modelled in 2018 and 2023, and the percentage of area covered by trees was measured in 2015. We used the residential history provided by the Digital and Population Data Services Agency, Finland between birth and 2020 to merge the urban physical exposures by EUREF-FIN geocodes. Exposures available in 2018 or 2015 were merged with residential addresses of participants in 2018 or 2015, while exposures available in 2023 were merged with residential addresses in 2020. Therefore, the exposures should be regarded as the exposures in 2015, 2018, or 2020 in the term of temporality.

#### 2.2.3. Other measures

Five sociodemographic variables were identified *a priori*: sex (categorical, female vs. male), age (continuous, year), work (categorical, not working or other situation vs. currently work), education (categorical, post-secondary or lower vs. bachelor/equivalent or above), and living status (categorical, with a spouse or a partner vs. with a spouse or a partner and child(ren), vs. other situation). The latter three were self-reported at the early midlife follow-up. Sex was based on the register information obtained when the cohort was established, while age was computed from the difference between the date of response and the date of birth. There were another three behavioral variables: illicit substance use (categorical, never vs. at least once), ever smoker (smoked over 100 cigarettes lifetime) (categorical, no vs. yes), and alcohol drinking (categorical, monthly or less or even never vs. 2–4 times a month or more), inquired also at the early midlife follow-up. Adult leisure-time physical activity was associated to most of the sociodemographic and behavioral variables, as shown in previous research (Abu-Omar et al., 2021; Poortinga, 2007; Samdahl and Jekubovich, 1997; Thompson et al., 2020).

To depict the social environment, four neighborhood social variables at the postal code level were derived from Statistics Finland in 2018: the proportion of resident living alone (single household), of residents with the lowest education level, of residents with the lowest income quartile, and of unemployed residents. A neighborhood deprivation score was generated from the latter three social variables (Kivimäki et al., 2020). We first standardized the three variables to z-scores, and their mean value was the deprivation score. Using a median split, we then categorized neighborhoods where participants lived in 2018 into two levels: low- and high-deprived. Thus, the two neighborhood social variables: the proportion of resident living alone and deprivation level were merged via residential history in 2018 too.

### 2.3. Analysis

#### 2.3.1. Data processing

After excluding those people who did not have information on leisure-time physical activity, sociodemographic, behavioral, and neighborhood-level social variables, 394 twin individuals resident in these urban areas were included in this study. Given that there were only 44 twin pairs with both cotwins satisfying the inclusion criteria, we did not consider zygosity as a covariate and did not perform any pairwise twin analysis. The distribution of sociodemographic and behavioral variables among included and excluded participants are presented in Supplemental Table 3. There were significant differences between included and excluded participants in education, illicit substance use, and alcohol drinking.

#### 2.3.2. Clustering analysis

The k-prototypes cluster analysis was employed to distinguish distinct patterns in the urban environment. It combines dissimilarity measures from both k-means and -modes algorithms for mixed types of exposures, and has shown to have a good performance (Huang, 1998; Preud’homme et al., 2021). Continuous exposures were standardized by standard deviation (SD). All 145 urban physical exposures were included in the clustering algorithms. The Silhouette method was used to pre-specify the number of clusters (Al- Zoubi and Rawi, 2008). One-step imputation within the algorithm was applied for missing values (Aschenbruck et al., 2023). Since k-prototypes cluster analysis is sensitive to outliers, the principal component analysis (PCA) of mixed data was conducted before. Three participants whose first or second principal components (PCs) fell outside the range of five standard deviations were identified as outliers (Jolliffe, 2002) and excluded from the cluster analysis.

Next, hierarchical linear regression was performed for the relationship between the urban cluster and leisure-time physical activity measures with three adjustment plans for covariates: 1) sociodemographic variables, 2) sociodemographic and behavioral variables, and 3) sociodemographic, behavioral, and neighborhood social variables. The cluster effect of sampling based on families of twin pairs was controlled by the robust standard error.

#### 2.3.3. Machine learning analysis

Before exploring the complexity within the urban environment via a pluralistic analysis platform, generalized linear regression models with the robust standard error were repeatedly performed between each leisure-time physical activity measure (total LTPA, LTPA, and commuting activity) and each urban physical exposure (missing values were imputed). The exposures were not placed in a single regression model. The *a priori* significant threshold of 0.01 was used to select noteworthy candidates and acknowledge the correlation between exposures. Dimensional reduction increases the model stability of subsequent analysis.

Then, we performed the eXtreme Gradient Boosting (XGBoost) model to assess the importance of urban physical exposures on each leisure-time physical activity measure, uncover interactions, and identify nonlinear relationships (Chen and Guestrin, 2016). It is an optimized distributed gradient boosting library designed for efficient and scalable training of machine learning models, with gradient-boosted decision trees algorithm (Chen and Guestrin, 2016). The hyperparameters were tuned through the 5-fold cross-validation grid search (Yu and Zhu, 2020). The participants were randomly split into training and testing subsets in a ratio of 3:1. The model performance was evaluated by root-mean-square error (RMSE). Selected urban physical exposures, sociodemographic, behavioral, and neighborhood social variables were included in the model. After hyperparameter tuning, the model was repeated two additional times with different seeds for result robustness. Due to the lack of counterfactual design, the current construction of XGBoost models did not consider any causal inference.

To increase model transparency, the SHapley Additive exPlanations (SHAP) value was used to interpret and visualize the results from the XGboost model, which featured the exposures’ importance on the outcome based on the cooperative game theory (Lundberg and Lee, 2017). Its direction suggests the effect’s direction on prediction, leading the model to predict either a higher or lower value of outcomes. Its magnitude is a measure of how strong the effect is. We quantified pairwise interaction SHAP values between included variables and summed their absolute value of all participants, with a high value indicating a strong interaction and synergistic effect (Lundberg et al., 2020). Additionally, Group-Lasso INTERaction-NET was performed for interaction to compare with the XGBoost’s result (Lim and Hastie, 2015).

#### 2.3.4. Sensitivity analysis

Due to missing values in urban physical exposures, we additionally performed sensitivity K-prototype cluster analysis and repeated generalized linear regression models between each urban physical exposure and each outcome, after removing participants with missing values (n = 13).

Then, we used PCA of mixed data again, as feature extraction, to transform the high-dimensional exposome data (all 145 exposures) into informative, compact indices. We extracted the top n PCs which explained over 80 % of the total variance. We then preformed XGBoost models including selected PCs, sociodemographic, behavioral, and neighborhood social variables for all three outcomes.

## 3. Results

### 3.1. Description of participants

Of the 394 included participants (mean age: 37, SD: 1.5) (Table 1), more individuals were female (55 %). Altogether, 87 %, 79 %, and 38 % of participants were employed, had at least bachelor-level education, and lived with a spouse/partner and child(ren), respectively. In their early midlife, more than half of the participants drank alcohol at least 2–4 times a month (58 %), but fewer were ever smoker (45 %) or had used illicit substances such as marijuana at least once (48 %). Before log-transformation, the means of total LTPA, LTPA, and commuting activity (unit: MET hours/day) were 5.4 (SD: 4.7), 4.3 (SD: 4.4), and 1.1 (SD: 1.0), respectively. After log-transformation, Spearman correlations between total LTPA and LTPA, between total LTPA and commuting activity, and between LTPA and commuting activity were 0.9, 0.3, and 0.1, respectively.

### 3.2. Results from clustering and hierarchical regression

**Fig. 2.**
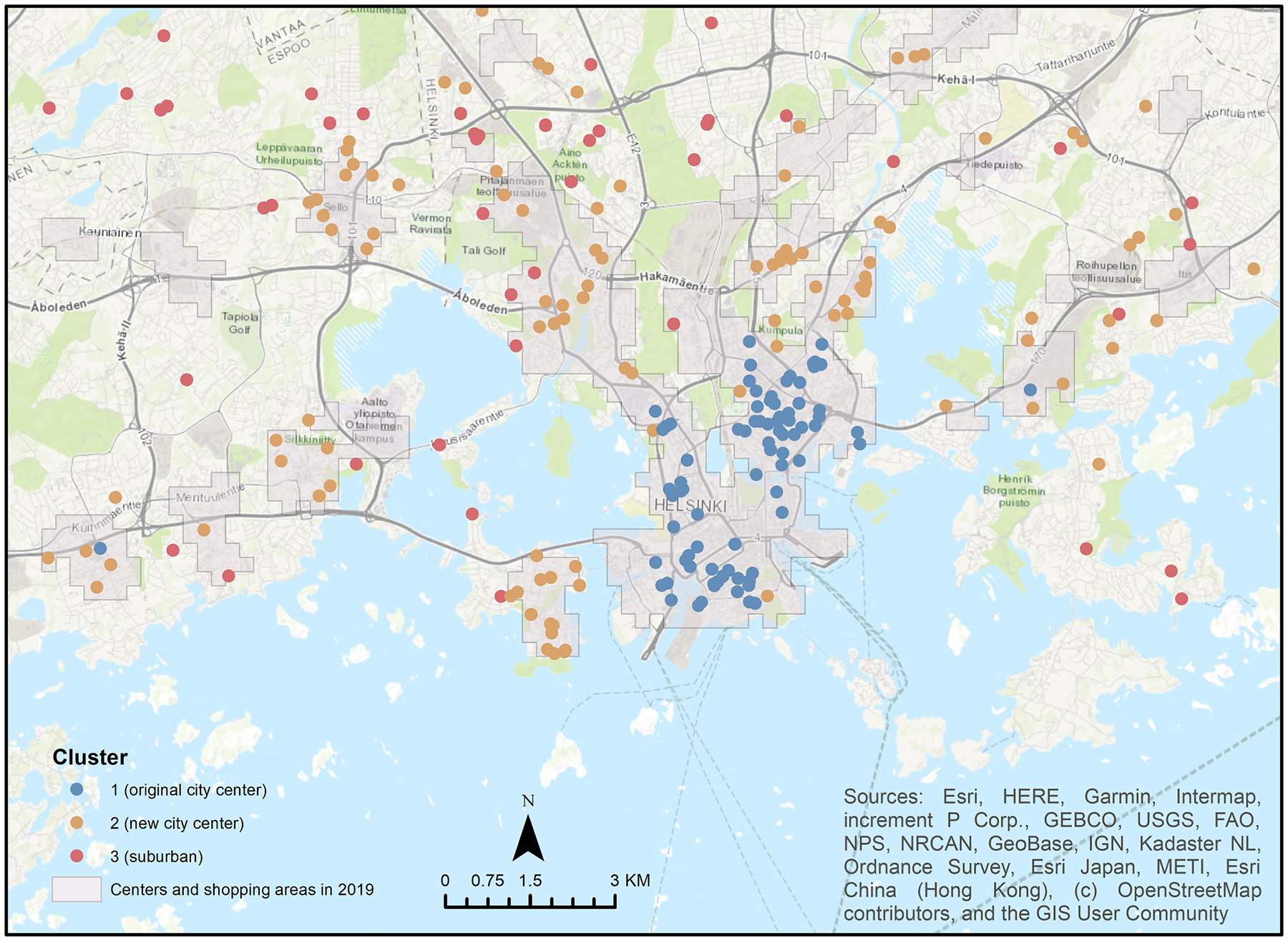
Twin participants’ residence in the Helsinki and Espoo area in 2018 colored by cluster Note: The gray layer shows centers and shopping areas in 2019.

The Silhouette method identified the optimal number of clusters to be three (largest Silhouette index, total within-cluster sum of squares: 42323.69). Using the map of Helsinki and Espoo and the spatial layer of centers and shopping areas in 2019 from the community structure monitoring system, Finnish Environment Institute (Viinikka et al., 2023), we classified Cluster 1, 2, and 3 as the “Original city center”, “New city center”, and “Suburban” clusters, respectively, based on the participants’ residence in 2018, as the urban cluster variable (Fig. 2). The naming process was solely based on visual observation.

After fully adjusting for sociodemographic, behavioral, and neighborhood social variables, compared to participants who lived in the “original city center” cluster, participants who lived in the “suburban” cluster were associated with significantly lower log-transformed scores of total LTPA (beta: −0.12, 95 % CI: −0.22, −0.03) and LTPA (beta: −0.16, 95 % CI: −0.28, −0.05) (Table 2). The effect sizes did not change substantially after adjustment of sociodemographic variables only and adjustment of both sociodemographic and behavioral variables. Regardless of adjustment plans, there was no significant association between the urban cluster and commuting activity (Table 2). There was no significant difference in any outcome between participants who lived in the “suburban” and “new city center” clusters. The powers of full-adjusted models of total LTPA, LTPA, and commuting activity were all 1.0.

### 3.3. Results from XGBoost

Based on the repeated generalized linear regression, there were 25 urban physical exposures significantly associated with total LTPA and 24 with LTPA (Supplemental Table 4). No urban physical exposure met the threshold p-value of 0.01 for association with commuting activity (Supplemental Table 4), so there was no XGBoost analysis for it.

**Fig. 3.**
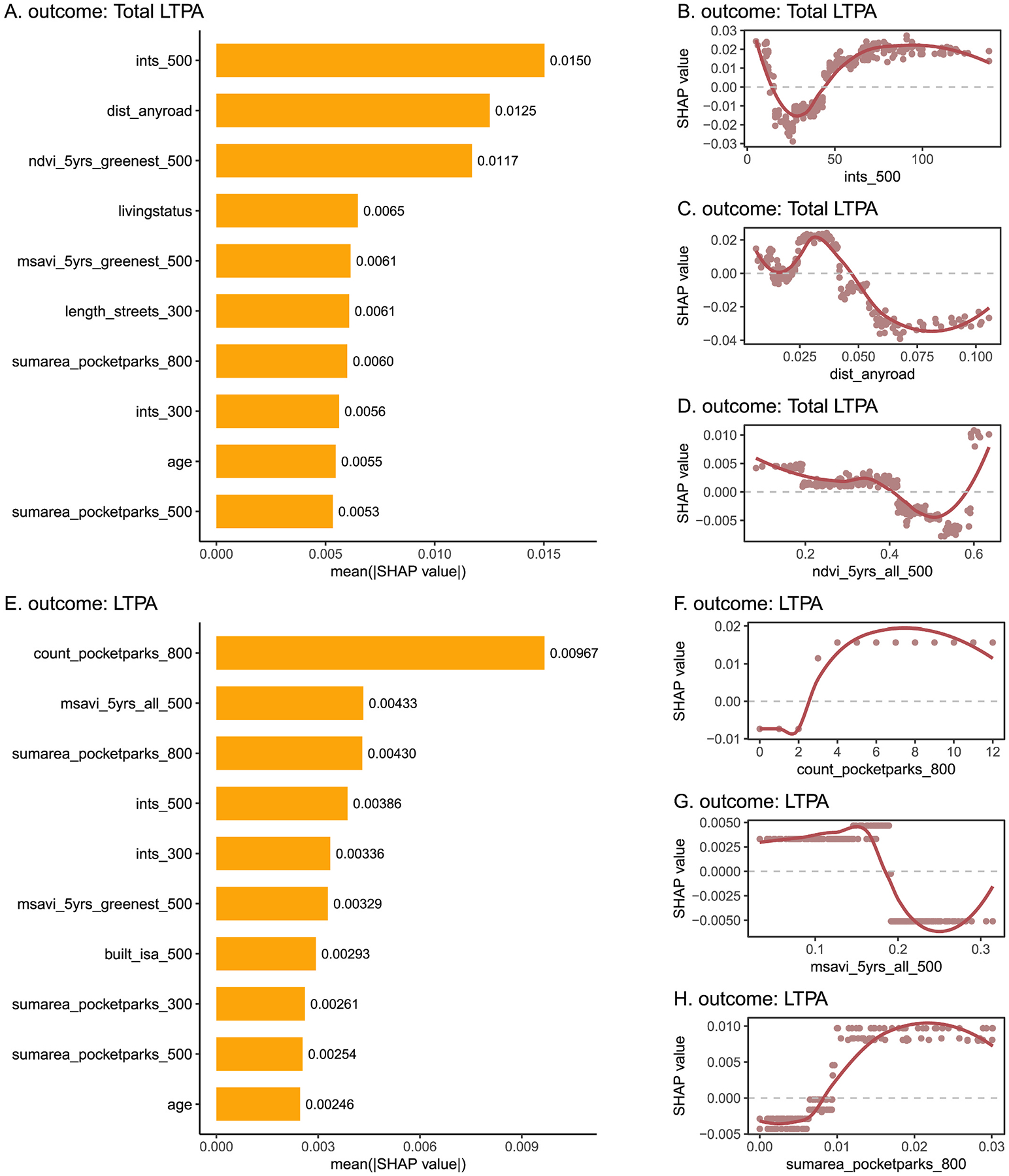
Results of XGBoost models for total leisure-time physical activity (total LTPA) and leisure-time physical activity without commuting activity (LTPA) Note: The SHAP bar plots show the influence of each variable: total LTPA (a) and LTPA (e). The SHAP dependence plots show how a single individual influences the XGboost prediction on total LTPA (b, c, d) and LTPA (f, g, h). ints_500 is the count of any type of road junctions within a 500 m buffer; sumarea_pocketparks_800 is the total area of all interconnected pocket parks within an 800 m walking distance; ndvi_5yrs_all_500 is the 5-years moving average of Normalized Difference Vegetation Index within a 500 m buffer during whole year; count_pocketparks_800 is the count of pocket parks within an 800 m walking distance. Abbreviation: leisure-time physical activity (LTPA); SHapley Additive exPlanations (SHAP).

In the XGBoost model of total LTPA including selected urban physical exposures, sociodemographic, behavioral, and neighborhood social variables, the top three important urban physical exposures were the count of any type of road junctions within a 500 m buffer (ints_500), the distance to the closest road (regardless of road type) (dist_anyroad), and the 5-years moving average of Normalized Difference Vegetation Index (NDVI), an indicator of general greenness, within a 500 m buffer around the home during whole year (ndvi_5yrs_all_500) (Fig. 3A). In dependence plots, a U-shaped relationship was observed between the SHAP value and the count of any type of road junctions within a 500 m buffer (Fig. 3B). When the count was between 15 and 50, it predicted a lower log-transformed total LTPA (negative SHAP values). If it was lower or higher than the range, it predicted a higher log-transformed total LTPA (positive SHAP values). Likewise, there was a non-linear relationship between the distance to the closest road and the SHAP value, with both W-shaped patterns (Fig. 3C). In Fig. 3D, when the 5-years moving average of NDVI within a 500 m buffer during whole year was below 0.2, the SHAP value was approximately 0.005. When it was between 0.2 and 0.4, SHAP values ranged from 0.001 to 0.002. When it was over 0.4, SHAP values became negative, but when it was further over 0.6, SHAP values turned positive again.

In the XGBoost model of LTPA (Fig. 3E), the top three most important urban physical exposures were the count of pocket parks within an 800 m walking distance (count_pocketparks_800), the 5-years moving average of Modified Soil Adjusted Vegetation Index (MSAVI), another indicator of general greenness, within a 500 m buffer around the home during whole year (msavi_5yrs_all_500), and the total area of all interconnected pocket parks within an 800 m walking distance (sumarea_pocketparks_800). There was a shift from negative to positive predictions of log-transformed LTPA, when the count of pocket parks within an 800 m walking distance exceeded two (Fig. 3F). For the 5-years moving average of MSAVI within a 500 m buffer around the home during whole year, SHAP values were positive below 0.018 and negative above 0.18 (Fig. 3G). Generally, when the total area of all interconnected pocket parks within an 800 m walking distance was below 0.01 km^2^, SHAP values were negative, and when it was over 0.01 km^2^, SHAP values were positive. Fig. 3G further suggested that the exposure looked like a four-group categorical variable.

Supplemental Fig. 2 displays pairwise SHAP interaction values in the XGBoost model of total LTPA, and there was some pairwise interaction between urban physical exposures but with low interaction SHAP value. Similarly, the XGBoost model of LTPA (Supplemental Fig. 3) indicates less interactions also with very low values. Group-Lasso INTERaction-NET models also did not capture any strong pairwise interaction for neither physical activity measure analyses.

The learning curves in training and testing subsets of models of total LTPA and LTPA are presented in Supplemental Fig. 4. For the XGBoost model of total LTPA, the RMSE is 0.23 in the training subset and 0.29 in the testing subset. In the two extra tests, the training RMSEs are both 0.24 and the testing RMSEs are 0.27 and 0.29. Comparing the reported test with two extra tests, the importance rank varied, but the count of any type of road junctions within a 500 m buffer was always the most or third most important (Supplemental Table 5). For the XGBoost model of LTPA, the RMSE is 0.32 in both training and testing subsets. In the two extra tests, the training RMSEs are both 0.31 and the testing RMSEs are 0.33 and 0.34. The importance rank also varied between reported results and two extra tests, but the most important urban physical exposure, the count of pocket parks within an 800 m walking distance, was in the top two in one extra test (Supplemental Table 6).

### 3.4. Sensitivity analysis

After excluding 13 participants with missing values in some urban physical exposures, the Silhouette method identified two clusters. In the following fully adjusted linear regression models, no significant differences in any of the leisure-time physical activity measures were found between the clusters. Repeated generalized linear regression analyses revealed 25 urban physical exposures significantly associated with total LTPA, consistent with the analysis using imputed data, and 26 exposures with LTPA, two more than the analysis with imputed data. Still, no urban physical exposure reached the 0.01 P-value threshold for association with commuting activity.

A total of 28 PCs explained over 80 % of the total variance of 145 urban physical exposures. The results of XGBoost models of total LTPA, LTPA, and commuting activity, which included PCs were presented in Supplemental Fig. 5. PC1 was the most important to predict both total LTPA and LTPA, and the vegetation indices and the percentage of buildup areas had high coefficients on PC1. PC2 and PC15 were the second important PCs to predict total LTPA and LTPA, respectively. PC25 and PC27 were the top two important PCs to predict commuting activity.

## 4. Discussion

We used clustering analysis and XGBoost to simultaneously and comprehensively study the effect of 145 urban physical exposures on leisure-time physical activity in 394 Finnish adults in their early midlife. Through transforming the high-dimensional exposure data into informative and compact cluster index, we have identified three clusters named: “original city center”, “new city center”, and “suburban”, based on the visual observation from the map. We found people living in suburban areas had a lower level of physical activity in leisure time compared to those living in the original city center. There was no difference between “original city center” and “new city center” clusters. XGBoost models revealed a complex relationship between the urban physical exposome and leisure-time physical activities, in which important exposures showed non-linearity and looked like threshold variables. More road junctions and shorter distances to the closest road correlated with higher levels of total LTPA (i.e. the higher mean MET hours per day). NDVI within a 500 m buffer below 0.4 or over 0.6 was correlated with higher levels of total LTPA, as well. Moreover, higher amounts of vegetation greenness (indicated by MSAVI) and smaller and less pocket parks within 800 m walking distance were associated with lower levels of LTPA. Comparing results of total LTPA and LTPA, the road characteristics became more important when considering commuting activity in leisure time. We did not find any considerable interaction between urban physical exposures contributing to leisure-time physical activities. These findings were correlational without further causal assessment.

Previous research has documented the relationship between different levels of urbanization and physical activity among the urban residents but with inconsistent findings regarding the direction of effects. A cross-sectional study in Shanghai, China with 327 respondents (mean age: 40) similarly reported higher leisure-time physical activity among downtown residents compared to suburban dwellers, which adhered to our findings (Zhou et al., 2013). A Finnish study indicated that children living in urban areas had a lower risk of overweight and obesity, a consequence of active physical activity (Mäki et al., 2023). Significant results were also found for transportation activities in this Chinese study, while we found a null result (Zhou et al., 2013). A Canadian study showed that the physical activity level was higher in urban than in suburban among adolescents from schools in lower socio-economic areas (Shearer et al., 2012). Nevertheless, a systematic review suggested that children and teenagers who live in suburban areas were more physically active than in rural and urban areas (Sandercock et al., 2010), and, similar to the Shanghai study above, a nationwide study in China showed that rising urbanization correlates with longer commuting times among adults (mean age: 45) (Zhu et al., 2017). In US, only male adolescents living in urban areas engaged in more moderate-to-vigorous physical activity than those living in suburban areas (Moore et al., 2014). The inconsistency between literature and our findings may be due to different population characteristics, sports cultures, country contexts, urban planning, or urbanicity definitions. Instead of a pre-definition of (sub)urban areas by governmental guidelines, we used an unsupervised data-driven clustering method to determine heterogeneous urban environments within urban areas reflecting real-life exposure modes and accounting for correlation, additive, and mixture effects (Guillien et al., 2021).

XGBoost models ranked the elements of road characteristics, greenspaces, and pocket parks, as strongly associated with leisure-time physical activities among early midlife adults. More road junctions and shorter distances to the closest road suggest better connectivity and accessibility, creating a more convenient environment for people to walk or bike to their destinations, A review of qualitative studies indicated access to local destinations and active transportation correlated with physical activity among adults (Salvo et al., 2018). A Finnish study found that the density of intersections, defined as the junction of a minimum of three roads, was positively associated with the number of physical activity bouts and the level of moderate to vigorous physical activity among older adults (Keskinen et al., 2020). Zang et al. (2022) identified the intersection density, as well as streetscape greenery, as the most important physical exposure contributing to light physical activity among older adults, by random forest models. However, the relationship between street connectivity, involving the number of intersections, and physical activity in all age groups of adults varied across different buffer areas in urban environments, suggesting the complexity of urban living environments (McGinn et al., 2007). Connectivity were also shown to be associated with transportation (McCormack and Shiell, 2011), although we did not find any important factors to commuting activities, the role of roads became more prominent when commuting activities were included in leisure-time physical activities. Where the association of greenspace with physical activity is relatively inconsistent (Browning et al., 2022), our findings show an association in which surrounding greenness is positively associated with LTPA up until a threshold of 0.4 NDVI, with higher NDVI relating to lower LTPA. High levels of greenspace might reflect suburban living to some extent, and other greenspace indicators, such as accessibility, were not prominent. The relationship between greenspace and physical activity could be moderated by the level of urbanization (Browning et al., 2022). Other studies have similar findings on the threshold effect. For example, the positive association of physical activity with multiple greenspace uses indicators reached to peak when indicators were within a 600 m buffer (Cardinali et al., 2024). Zang et al. (2022) also found that streetscape greenery had a positive effect on light physical activity when it ranged from 0.12 to 0.15 point, corresponding to a low level of visible greenery. Besides, another Chinese study also identified the 0.4 NDVI, corresponding to areas with sparse to moderate vegetation, as the turning point for its association with self-rated health among the old population (Huang et al., 2022), and self-rated health closely correlated with physical activity (Guan, 2022). For pocket parks, a natural experimental study in low-income American neighborhoods found a higher level of leisure-time exercise among middle-aged residents after pocket parks were constructed (Cohen et al., 2014). Users of pocket parks, defined as living within a 0.5 mile (~800 m) radius, had higher exercise levels than traditional park users (Cohen et al., 2014). Researchers further summarized that pocket parks were cost-effective for promoting physical activity in inner-city areas (Cohen et al., 2014). A study in Chongqing, China, utilizing interviews on conceptual understanding of park images, revealed that the environmental characteristics of pocket parks contributed to a restorative effect involving entertainment activities and relief (Peng et al., 2023). Noteworthy, a recent Chinese study using Light Gradient-Boosting Machine model found that recreational facilities were the most important factor for walking behavior in old adults but the number of parks was the least important among 11 factors, highlighting the specific effect driven by the content inside parks or recreation areas (Yang et al., 2024). This annotation on benefit threshold may provide more precise guidance on urban planning, but more replication and exploration is needed to validate these findings for country- or European-wide implications.

The identified exposures may be elaborated by factors such as accessibility, opportunities, and aesthetic attributes individual felt or perceived from the better availability and quality of green recreational spaces and improved connectivity. A systematic review demonstrated that accessibility to facilities, the presence of sidewalks, and aesthetic features positively correlate with physical activity participation, including both leisure and commuting activities, among adults (Choi et al., 2017). Perceived neighborhood environmental factors such as street connectivity were correlated with moderate to vigorous physical activity and commuting activity (Carlson et al., 2018). Social cognitive theory further suggests that individuals are shaped by and respond to their social and built environments. They also engage in forethought, self-regulation, self-reflection, vicarious learning, and innovation through imagination and communication (Bandura, 1986). Environmental factors were correlated with self-efficacy beliefs (Beauchamp et al., 2019), which have been consistently linked to physical activity (Young et al., 2014). Among adolescents, self-efficacy for overcoming barriers was shown to mediate the effect of perceived equipment accessibility on physical activity in a cross-sectional study (Motl et al., 2005). While our analyses used objective environmental measures, future studies integrating perceived environmental and interpersonal factors could provide deeper insights into the mechanisms underlying behavior for informing effective interventions (Beauchamp et al., 2019).

Besides its strength, this study is not without limitations. First, the sample size was relatively small compared to other exposome studies. Although the sample size for K-prototype clustering (over 10 times the number of clusters) and subsequent regression seems to be adequate, inconsistency in additional XGBoost models highlights the need for a larger sample. Additionally, due to the complexity of the large-dimensional exposome set, the modest sample size made capturing relatively small interactions more challenging. The twin design was also not employed to control for the unobserved individual heterogeneity (confounding). Second, only participants from the five largest cities in Finland were included, limiting the generalizability. The included participants represented 19 % of twins who have completed early midlife follow-up, while the five cities covered approximately 28 % of the total Finnish population. Besides, we did not include any participants living in rural areas. Not only the physical environment, but lifestyles may also differ between urban and rural areas. Therefore, the interpretation should be narrowed down to specific types of cities. Third, urban physical exposures were based on residential addresses, which overlook dynamic human behaviors outside the home, leading to measurement errors. In addition, the used residential geocodes corresponded to participants’ residences in 2017, 2018, or 2020, without accounting for how long they lived at those addresses. Measurement errors could skew our identification of key determinants, as exposures with larger errors might show weaker associations and be classified as less influential, even if they are actually more important than those identified as most influential. More granular and accurate estimations of exposure and behavior could facilitate the exploration in the dynamic interaction between the environment and human behavior (Sonnenschein et al., 2022). Fourth, some exposures were available in 2023 but merged with the address in 2020, posing a temporality issue. The relatively slow urban renewal and construction in Finland reduced the concern (Evers et al., 2024). Fifth, missing values in exposures may introduce bias. Excluding participants with missing values altered the optimal number of clusters, while the number of significant associations between exposures and outcomes remained similar to the number based on imputed data. Given that only about 3 % of participants had missing values, the effect is likely modest, but caution is still warranted. Sixth, leisure-time physical activity was self-reported. MET values used in the current study were given by the Compendium of Physical Activity being estimates of the resting oxygen consumption of an average man, even though the energy expenditure is always related to factors as body weight, body composition and movement efficiency (Ainsworth et al., 2011; Herrmann et al., 2024). The device-based measurement of leisure-time physical activity would have been more accurate. However, the validity of leisure-time physical activity questions used in Finnish twins has been demonstrated (Leskinen et al., 2009; Waller et al., 2008). Seventh, the naming of clusters may introduce subjectivity and oversimplify complex urban environmental patterns, even though our unsupervised clustering method identified novel structures. The agnostic, data-driven nature of the results should be carefully considered in the interpretation. Eighth, there was no causal assessment. Unmeasured confounding (although a wide range of exposures and covariates included), reverse relationships, and other issues remain as concerns; outputs may be over- or under-estimated. In short, a complex association was observed, but due to the correlational and observational nature of the study and these mentioned limitations, firm causal conclusions cannot be drawn, and further endeavor is needed to explore underlying mechanisms with a rigorous design.

## 5. Conclusion

This study employed two analytical approaches to explore the intricate correlational relationship between the urban physical exposome and leisure-time physical activity in early midlife in Finland. Clustering analysis revealed three heterogeneous patterns of urban environments. Living in suburban areas was associated with lower levels of leisure-time physical activity than in original city center areas. XGBoost models identified pocket parks, road characteristics, and greenspaces as influential factors with non-linear relationships, which behaved like threshold variables. Given limitations in causality, sample size, generalizability, and measurement granularity, we call for further studies in other settings to replicate our analyses. We still advocate presenting this evidence to stakeholders and policymakers to consider the complexity in developing tailored interventions on some urban features to achieve higher cost-effectiveness by focusing on the most influential determinants and their optimal ranges in addressing the challenge of the physically inactive lifestyle in our rapidly urbanizing world.

## Supplementary Material

1

## Figures and Tables

**Table 1:** Characteristics of sociodemographic, behavior, and neighborhood social variables (participants n = 394).

Characteristics	N. (%)/Mean (SD)
Sex	
Male	179 (45.4)
Female	215 (54.6)
**Work**	
Not working or other situation	51 (12.9)
Currently work	343 (87.1)
**Education**	
Post-secondary or lower	84 (21.3)
Bachelor/equivalent or above	310 (78.7)
**Living status**	
With a spouse or a partner	118 (30.0)
With a spouse or a partner and child(ren)	151 (38.3)
Other situation	125 (31.7)
**Age (years)**	37.1 (1.5)
**Illicit substance use**	
No	204 (51.8)
Yes	190 (48.2)
**Ever smoker (smoked over 100 cigarettes lifetime)**	
No	216 (54.8)
Yes	178 (45.2)
**Alcohol**	
Monthly or less, or even never	164 (41.6)
2–4 times a month or more	230 (58.4)
**Deprivation level**	
Low	225 (57.1)
High	169 (42.9)
**The proportion of single households in the neighborhood**	50.0 (10.8)

**Table 2:** Results of the linear regression between the urban cluster and physical activity measures.

Outcome (log-transformed)	Characteristics	Model 1^a^		Model 2^b^		Model 3^c^	
		Beta (95 % CI)	R^2^	Beta (95 % CI)	R^2^	Beta (95 % CI)	R^2^
**Total LTPA**	**Urban cluster**						
	1 (original city center)	Ref.	0.08	Ref.	0.08	Ref.	0.09
	2 (new city center)	−0.06 (−0.13, 0.01)		−0.06 (−0.13, 0.01)		−0.05 (−0.13, 0.02)	
	3 (suburban)	−0.13 (−0.21, −0.06)*		−0.13 (−0.21, −0.05)*		−0.12 (−0.22, −0.03)*	
**LTPA**	**Urban cluster**						
	1 (original city center)	Ref.	0.08	Ref.	0.08	Ref.	0.08
	2 (new city center)	−0.07 (−0.15, 0.01)		−0.06 (−0.15, 0.02)		−0.07 (−0.16, 0.03)	
	3 (suburban)	−0.16 (−0.25, −0.07)*		−0.16 (−0.25, −0.06)*		−0.16 (−0.28, −0.05)*	
**Commuting activity**	**Urban cluster**						
	1 (original city center)	Ref.	0.03	Ref.	0.06	Ref.	0.06
	2 (new city center)	−0.01 (−0.06, 0.03)		−0.01 (−0.05, 0.04)		0.00 (−0.04, 0.05)	
	3 (suburban)	−0.03 (−0.08, 0.02)		−0.03 (−0.08, 0.02)		−0.01 (−0.07, 0.05)	

* P < 0.05.

a Adjusted for age, sex, education, work, and living status.

b Based on model 1, additionally adjusted for smoking, alcohol drinking, and illicit substance use.

c Based on model 2, additionally adjusted for neighborhood deprivation level and the proportion of single households in the neighborhood.

## Data Availability

The FinnTwin12 data are not publicly available due to the restrictions of informed consent. However, the FinnTwin12 data are available through the Institute for Molecular Medicine Finland (FIMM) Data Access Committee (DAC) (fimm-dac@helsinki.fi) for authorized researchers who have IRB/ethics approval and an institutionally approved study plan. To ensure the protection of privacy and compliance with national data protection legislation, a data use/transfer agreement is needed, the content and specific clauses of which will depend on the nature of the requested data. Requests will be addressed in a reasonable time frame (generally two to three weeks), and the primary mode of data access is by either personal visit or remote access to a secure server. Code for major analyses is available at https://github.com/doge73/city_urban_PA.

## Data availability

Data will be made available on request.

## Appendix A. Supplementary data

Supplementary data to this article can be found online at https://doi.org/10.1016/j.healthplace.2025.103495.
